# Ethanol Modulates the Spontaneous Complex Spike Waveform of Cerebellar Purkinje Cells Recorded *in vivo* in Mice

**DOI:** 10.3389/fncel.2017.00043

**Published:** 2017-02-28

**Authors:** Guang-Jian Zhang, Mao-Cheng Wu, Jin-Di Shi, Yin-Hua Xu, Chun-Ping Chu, Song-Biao Cui, De-Lai Qiu

**Affiliations:** ^1^Key Laboratory of Cellular Function and Pharmacology of Jilin Province, YanBian UniversityYanji City, China; ^2^Department of Pain, Affiliated Hospital of Yanbian UniversityYanji City, China; ^3^Department of Osteology, Affiliated Hospital of Yanbian UniversityYanji City, China; ^4^Department of Physiology and Pathophysiology, College of Medicine, Yanbian UniversityYanji City, China; ^5^Department of Neurology, Affiliated Hospital of Yanbian UniversityYanji City, China

**Keywords:** ethanol, cerebellar Purkinje cell, *in vivo* whole-cell patch-clamp recording, complex spike (CS), after-hyperpolarization (AHP), cannabinoids receptor 1 (CB1)

## Abstract

Cerebellar Purkinje cells (PCs) are sensitive to ethanol, but the effect of ethanol on spontaneous complex spike (CS) activity in these cells *in vivo* is currently unknown. Here, we investigated the effect of ethanol on spontaneous CS activity in PCs in urethane-anesthetized mice using *in vivo* patch-clamp recordings and pharmacological manipulation. Ethanol (300 mM) induced a decrease in the CS-evoked pause in simple spike (SS) firing and in the amplitude of the afterhyperpolarization (AHP) under current clamp conditions. Under voltage-clamp conditions, ethanol significantly decreased the area under the curve (AUC) and the number of CS spikelets, without changing the spontaneous frequency of the CSs or the instantaneous frequency of the CS spikelets. Ethanol-induced a decrease in the AUC of spontaneous CSs was concentration dependent. The EC_50_ of ethanol for decreasing the AUC of spontaneous CSs was 168.5 mM. Blocking N-methyl-D-aspartate receptors (NMDARs) failed to prevent the ethanol-induced decreases in the CS waveform parameters. However, blockade of cannabinoid receptor 1 (CB1) significantly suppressed the ethanol-induced effects on the CS-evoked pause in SS firing, amplitude of the AHP, spikelet number and the AUC of CSs. Moreover, a CB1 receptor agonist not only reduced the number of spikelets and the AUC of CSs, but also prevented the ethanol-induced inhibition of CS activity. Our results indicate that ethanol inhibits CS activity via activation of the CB1 receptor *in vivo* in mice, suggesting that excessive ethanol intake inhibits climbing fiber (CF)–PC synaptic transmission by modulating CB1 receptors in the cerebellar cortex.

## Introduction

The cerebellum integrates sensory and motor information and generates motor-related outputs that are involved in motor coordination, motor learning and the fine adjustment of voluntary movement. Cerebellar Purkinje cells (PCs) receive sensory and motor information through the mossy fiber–parallel fiber (MF–PF) and the climbing fiber (CF) pathways (Ito, 1984).

Each cerebellar PC receives a single CF input that originates from the inferior olive of the medulla (Ito, 1984). The CFs make synapses with PC dendrites that excite PCs by generating powerful excitatory postsynaptic potentials (EPSPs), called complex spikes (CSs; Simpson et al., 1996; Strata and Rossi, 1998). Because the CF–PC synapses consist of a large number of synaptic contact sites, and the release of glutamate at each CF terminal has high probability, CF–PC synaptic transmission is powerful and reliable (Dittman and Regehr, 1998; Hashimoto and Kano, 1998). Activation of the CF evokes a strong and irregular all-or-none CS that consists of a fast sodium spike and several spikelets (Ito, 1984). *In vivo*, CSs normally occur at low frequencies of about 1 Hz, but can increase to 11 Hz during nociceptive stimulation (Ekerot et al., 1987), and the instantaneous frequency of CS spikelets can reach up to 500 Hz (Maruta et al., 2007). CSs are thought to encode important information for cerebellar cortical functions, such as timing information, and the triggering of synaptic plasticity (Hansel et al., 2001; Ito, 2001). *In vivo*, CF discharges modulate the frequency and pattern of PC simple spike (SS) output by punctuating tonic activity with variable-duration pauses (Ebner et al., 1983; Barmack and Yakhnitsa, 2003; Cerminara and Rawson, 2004).

It was suggested that N-methyl-D-aspartate receptors (NMDARs) do not contribute to CF activation-mediated inward currents (Otis et al., 1997; Auger and Attwell, 2000). However, NMDARs are postsynaptically expressed at CF–PC synapses in adult mice and participate in the synaptic currents mediated by CF activity in PCs (Piochon et al., 2007). We recently found that extracellular application of NMDA enhances the waveform of spontaneous CS-evoked inward currents, suggesting that CF–PC synaptic transmission is enhanced by activation of NMDARs at CF–PC synapses (Liu et al., 2016). It is clear that CF activation evokes calcium spikes in primary dendrites and their branches (Llinas and Sugimori, 1980; Ross and Werman, 1987; Miyakawa et al., 1992). Therefore, CF–PC synaptic NMDARs might induce a phasic influx of Ca^2+^ with a spatiotemporal profile potentially different from that of Ca^2+^ elevation associated with CF-evoked CSs (Hartmann and Konnerth, 2005). In addition, CF-evoked dendritic Ca^2+^ spikes are related to calcium influx, which is partially mediated by P/Q-type voltage-dependent calcium channels in mammalian cerebellar PCs (Usowicz et al., 1992). Importantly, CF activity is required for the induction of long-term depression (LTD) and long-term potentiation (LTP) at PF–PC synapses, suggesting that CF signaling is involved in motor learning (Ito, 1984; Hansel and Linden, 2000; Coesmans et al., 2004).

The cerebellum is a target of the acute action of ethanol. Ethanol intoxication impairs cerebellar functions, including motor coordination, balance, behavior, speech and certain cognitive processes (Schmahman and Sherman, 1997; Botta et al., 2007). Ethanol-induced cerebellar dysfunction is considered to be mediated, at least in part, by the impairment of cerebellar neuronal function (Mameli et al., 2008). *In vitro*, low concentrations of ethanol increase the current injection-evoked SS firing rate, while high concentrations elicit a reduction in SS firing rates (Freund et al., 1993). Ethanol increases GABAergic transmission onto cerebellar PCs by enhancing calcium release from presynaptic internal stores and by increasing the intrinsic firing rate of molecular layer interneurons (MLIs) in rat cerebellar slices (Mameli et al., 2008). Furthermore, ethanol increases GABA release from MLIs onto PCs, resulting in an increase in the frequencies of spontaneous and miniature inhibitory postsynaptic currents, and a decrease in the amplitude of excitatory postsynaptic currents in cerebellar PCs (Mameli et al., 2008; Hirono et al., 2009; Wadleigh and Valenzuela, 2012).

We previously reported that high concentrations of ethanol significantly inhibit sensory stimulation-evoked responses in the cerebellar molecular (Cui et al., 2014) and granule cell (Wu et al., 2014) layers via activation of GABA_A_ receptors in urethane anesthetized mice, suggesting that high concentrations of ethanol impair sensory information processing in the cerebellar cortex. More recently, we found that ethanol dose-dependently inhibits facial stimulation-evoked outward currents by activating presynaptic cannabinoid receptor 1 (CB1) receptors via the PKA signaling pathway. These observations suggest that ethanol overdose impairs sensory information processing, at least in part, by inhibiting GABA release from MLIs onto PCs (Wu et al., 2016).

In this study, we investigated the effect of ethanol on spontaneous CS activity in PCs in urethane-anesthetized mice, using *in vivo* patch-clamp recording and pharmacological treatment. We found that ethanol induces a CB1 receptor-dependent inhibition of CS activity in cerebellar PCs, suggesting that excessive ethanol intake inhibits the transmission of peripheral afferent information to cerebellar PCs via activation of the CB1 receptor at CF–PC synapses.

## Materials and Methods

### Anesthesia and Surgical Procedures

The anesthesia and surgical procedures have been described previously (Chu et al., 2011b). In brief, the experimental procedures were approved by the Animal Care and Use Committee of Jilin University and were in accordance with the animal welfare guidelines of the U.S. National Institutes of Health. The permit number is SYXK (Ji) 2007-0011. Either male (*n* = 31) or female (*n* = 30) adult (6–8-week-old) HA/ICR mice were anesthetized with urethane (1.3 g/kg body weight i.p.). Mice were tracheotomized to avoid respiratory obstruction. On a custom-made stereotaxic frame, a watertight chamber was created and a 1–1.5 mm aperture was drilled on the skull to expose the cerebellar surface corresponding to Vermis VI-VII. The dura was carefully removed, and the brain surface was constantly superfused with oxygenated artificial cerebrospinal fluid (ACSF: 125 mM NaCl, 3 mM KCl, 1 mM MgSO_4_, 2 mM CaCl_2_, 1 mM NaH_2_PO_4_, 25 mM NaHCO_3_, and 10 mM D-glucose) with a peristaltic pump (Gilson Minipulse 3; Villiers, Le Bel, France) at 0.4 ml/min. Rectal temperature was monitored and maintained at 37.0 ± 0.2°C using body temperature equipment.

### Electrophysiological Recording and Histochemistry

*In vivo* whole-cell recordings from PCs (*n* = 46 cells) were performed with an Axopatch-200B amplifier (Molecular Devices, Foster City, CA, USA) in cerebellar cortical lobule Vermis VIb from 46/61 mice. Fifteen mice were failed to be obtained whole-cell recordings from PCs, and each of 46 mice was recorded one PC for further experiment. The signals of PC whole-cell recordings were acquired through a Digidata 1440 series analog-to-digital interface on a personal computer using Clampex 10.3 software. Patch pipettes were made with a puller (PB-10; Narishige, Tokyo, Japan) from thick-wall borosilicate glass (GD-1.5; Narishige). Patch electrodes (4–6 MΩ) contained a solution of the following composition (in mM): potassium gluconate 120, HEPES 10, EGTA 1, KCl 5, MgCl_2_ 3.5, NaCl 4, biocytin 8, Na_2_ATP 4, and Na_2_GTP 0.2 (pH 7.3 with KOH, osmolarity adjusted to 300 mOsm). The PCs whole-cell recordings from PCs were performed at depths 150–200 μm under pia mater membrane, and identified by regular spontaneous SS accompanied with irregular CS (Chu et al., 2011b), and confirmed by biocytin histochemistry (Chu et al., 2011a). The series resistances were in a range of 10–40 MΩ, compensated by 80%. Membrane potential and current were filtered at 2 kHz, digitized at 20 kHz. Spontaneous activity was calculated from a train of interspike intervals recorded for 100 s.

After the experiments, the whole brain was removed and fixed in 4% paraformaldehyde in 0.1 PBS (pH 7.4) at 4°C for 24 h. Slices were cut in the sagittal plane at 200 μm using a vibratome (NVSLM1, Campden Instruments LTD, Loughborough, England), and washed with PBS. The tissue was reacted overnight with an avidin-biotin complex (ABC Elite kit; Vector Laboratories, Burlingame, CA, USA) at 4°C. Finally, biocytin binding was visualized by 3,3^′^-diaminobenzidine tetrahydrochloride histochemistry.

### Chemicals and Drug Application

The reagents included N-(piperidin-1-yl)-5-(4-iodophenyl)-1-(2,4-di-chlorophenyl)-4-methyl-1H-pyrazole-3-carboxamide (AM251), blockade of endocannabinoid CB1 receptors; (R)-(+)-[2,3-dihydro-5-methyl-3-(4-morpholinylmethyl) pyrrolo[1,2,3-de]-1,4-benzoxazin-6-yl]-1-naphthalenylmethanone mesylate (WIN55212-2), CB1 receptors agonist; and D-(-)-2-Amino-5-phosphonopentanoic Acid (D-APV). All chemicals were purchased from Sigma-Aldrich (Shanghai, China). AM251 and WIN552121 were dissolved in ethanol (20 mM) for stock solution. All drugs were finally dissolved in ACSF, and applied directly onto the cerebellar surface by a peristaltic pump (0.5 ml/min) for 10 min. Consider of the effects of blood flow and cerebrospinal fluid, the chemicals were usually used *in vivo* at 5–10 times higher than that in brain slices. Therefore, we applied ethanol up to 300 mM, and D-APV up to 250 μM on the cerebellar surface.

### Data Analysis

The electrophysiological data were analyzed using Clampfit 10.3 software. Values are expressed as the mean ± SEM. One-way and repeated measures ANOVA followed by Tukey’s *post hoc* test (SPSS software; Chicago, IL, USA) were used to determine the level of statistical significance between groups of data. *P*-values below 0.05 were considered to indicate a statistically significant difference between experimental groups.

## Results

### Ethanol Inhibits Spontaneous CS Activity in Cerebellar PCs

**Figure 1:**
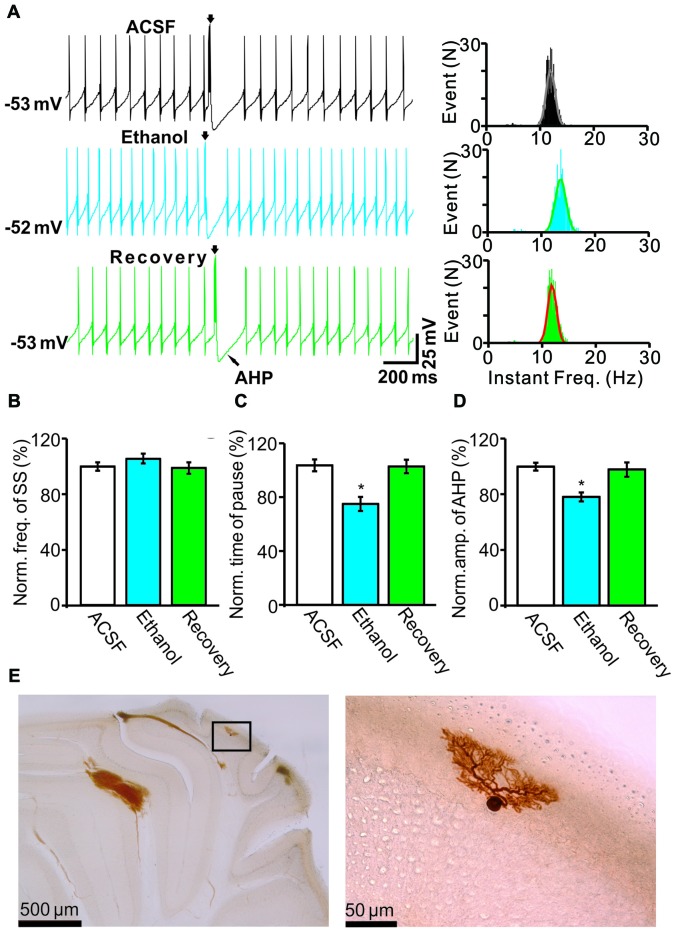
**Effects of ethanol on the spontaneous activity of Purkinje cells (PCs). (A)** Left, representative traces showing the spontaneous activities of a PC in the presence of artificial cerebrospinal fluid (ACSF), ethanol (300 mM) and washout. Right, histograms show the instantaneous frequency of simple spikes (SS). **(B,C)** Bar graphs show the effect of ethanol on the frequency **(B)** and the pause **(C)** of SSs. **(D)** Summary of data showing the normalized amplitude of the afterhyperpolarization (AHP) in the presence of ACSF, ethanol (300 mM) and washout. **(E)** The photomicrographs show the morphology of the cell, which is shown in **(A)**. The left column shows an overview of the location of the biocytin-labeled cell. The right column shows the detail of the biocytin-labeled cell. Note that ethanol significantly decreases both the amplitude of the AHP and the pause in SS firing. *n* = 8 cells per group. *P < 0.05, vs. ACSF.

**Figure 2:**
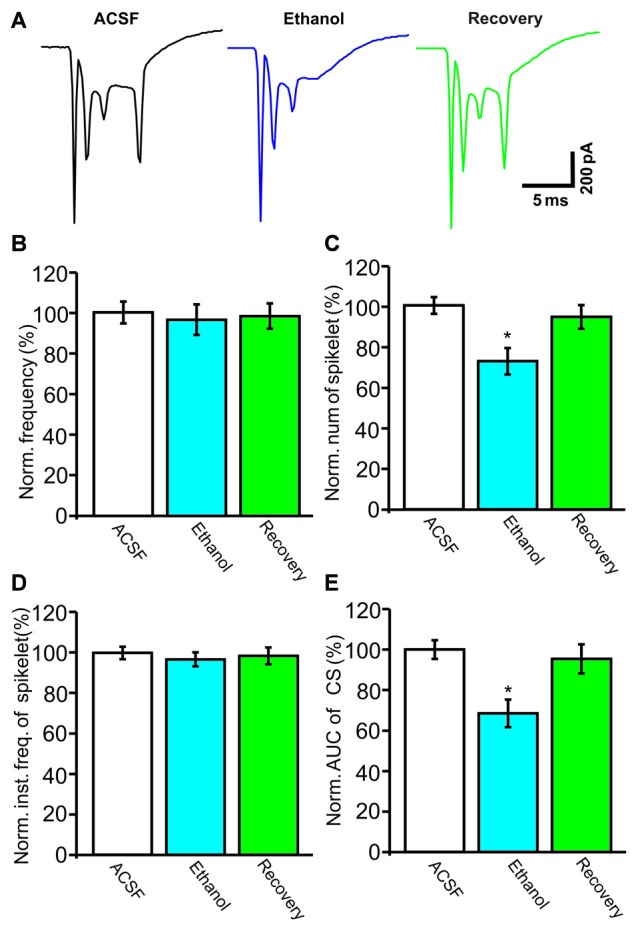
**Under voltage-clamp recording conditions, ethanol inhibits the spontaneous complex spike (CS)-evoked inward currents. (A)** Representative traces showing the properties of the CS-evoked inward currents in a PC in the presence of ACSF, ethanol (300 mM) and washout. **(B)** Bar graph shows the effect of ethanol on the frequency of CSs. **(C)** Pooled data showing the effect of ethanol on the number of CS spikelets. **(D)** Bar graph shows the effects of ethanol on the instantaneous frequency of spikelets. **(E)** Summary of data showing the normalized area under the curve (AUC) of CSs in ACSF, ethanol (300 mM) and washout. *n* = 8 cells per group. *P < 0.05, vs. ACSF.

**Figure 3:**
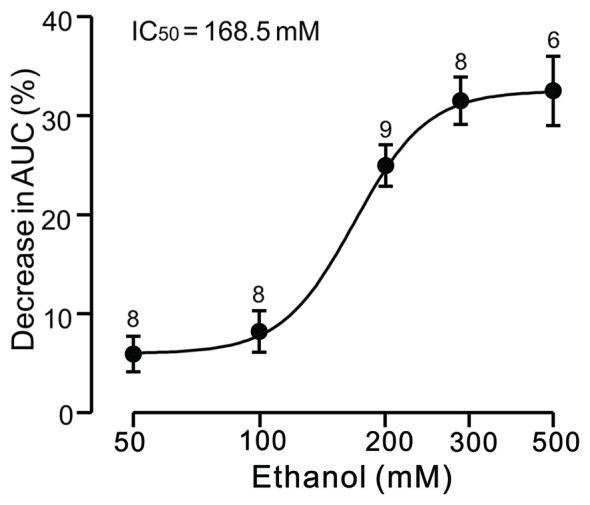
**Concentration-response curve showing the ethanol-induced decrease in the AUC of CS-induced inward currents in cerebellar PCs.** The IC_50_ value obtained from the curve is 168.5 mM. The number of recorded PCs tested for each concentration is indicated near the bars. Error bars indicate S.E.M.

Under current-clamp conditions (*I* = 0), cerebellar PCs exhibited regular SS firing at a mean frequency of 29.6 ± 0.4 Hz (*n* = 46 cells), and irregular CS firing at a mean rate of 1.1 ± 0.13 Hz (*n* = 46 cells), recorded in the cerebellar cortical vermis VIb. The number of spikelets was 2–5, and their mean frequency was 392.7 ± 5.1 Hz (*n* = 46 cells). In addition, CS evoked an afterhyperpolarization (AHP) potential with a mean amplitude of 16.3 ± 1.7 mV (*n* = 46 cells), accompanied with a pause in SS firing at 78.5 ± 2.1 ms (*n* = 46 cells). Application of ethanol (300 mM) did not significantly change the instantaneous frequency (Figure 1A) or the mean frequency of spontaneous SSs (Figure 1B). The normalized SS frequency was 105.2 ± 3.7% of baseline (99.2 ± 4.8%, *n* = 8 cells, *P* = 0.37; Figure 1B). However, the normalized pause in SS firing was 72.3 ± 5.0% of baseline (99.2 ± 4.8%, *n* = 8 cells, *P* = 0.0003; Figures 1A,C), and the amplitude of the CS-induced AHP was 78.2 ± 3.3% of baseline (ACSF: 97.9 ± 5.2%, *n* = 8 cells, *P* = 0.021; Figures 1A,D). Biocytin staining indicated that the recorded cell was a cerebellar PC (Figure 1E; Chu et al., 2011a). Under voltage-clamp conditions (*V*_hold_ = −70 mV), the spontaneous CS displayed strong inward currents with high frequency spikelets, followed by outward currents (Figure 2A). Ethanol (300 mM) did not change the frequency of spontaneous CS activity (Figures 2A,B) or the instantaneous frequency of CS spikelets (Figures 2A,D), but it decreased the number of spikelets and the area under the curve (AUC) of CS-evoked inward currents to 73.4 ± 6.8% of baseline (ACSF: 100.1 ± 4.9%, *n* = 8 cells, *P* = 0.016; Figures 2A,C) and 69.1 ± 7.5% of baseline (ACSF: 100.0 ± 5.1%, *n* = 8 cells, *P* = 0.008; Figures 2A,E), respectively. In addition, the ethanol-induced decrease in the AUC of CSs was dose-dependent (Figure 3). The AUC of CSs was significantly decreased by 50 mM ethanol (5.9 ± 3.6% decrease, *n* = 8 cells), with an IC_50_ of 168.5 μM. These results indicate that cerebellar surface application of ethanol produces a dose-dependent decrease in the SS pause and the amplitude of the AHP, without affecting SS firing rate *in vivo* in mice.

### Blockade of NMDARs does not Prevent the Ethanol-Induced Inhibition of CS Activity

**Figure 4:**
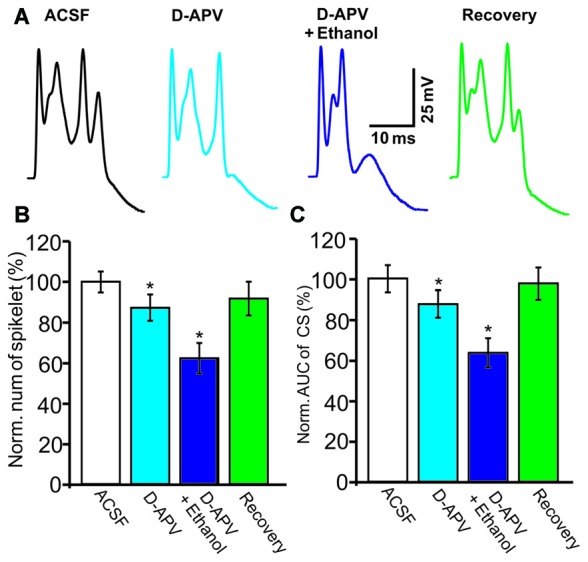
**The ethanol-induced inhibition of CS activity is persistent in the presence of an N-methyl-D-aspartate receptors (NMDAR) blocker. (A)** Representative traces showing the properties of the CS-evoked inward currents in the presence of ACSF, D-(-)-2-Amino-5-phosphonopentanoic Acid (D-APV; 250 μM) or a mixture of D-APV + ethanol (300 mM), and recovery. **(B,C)** Bar graphs showing the effect of ethanol on the spikelet number of CSs **(B)** and the AUC of CSs in the presence of ACSF, D-APV (250 μM) or a mixture of D-APV (50 μM) + ethanol, and recovery. *n* = 6 cells per group. *P < 0.05, vs. ACSF.

NMDARs are activated by CF stimulation and contribute to the CS waveform (Piochon et al., 2007; Renzi et al., 2007), and ethanol has been shown to reduce the CS afterdepolarization by modulating the NMDAR in cerebellar slices (He et al., 2013). Therefore, we examined whether ethanol inhibits the CS waveform by affecting NMDAR activity. After application of an NMDAR antagonist, D-APV (250 μM), the number of spikelets was reduced to 87.3 ± 6.5% of baseline (ACSF: 100.0 ± 4.9%, *n* = 8 cells, *P* = 0.028; Figures 4A,B) and the AUC of CSs was reduced to 87.6 ± 6.7% of baseline (ACSF: 100.1 ± 6.6%, *n* = 8 cells, *P* = 0.031; Figures 4A,C). However, D-APV failed to prevent the ethanol-induced inhibition of CS activity. After application of a mixture of D-APV and ethanol (300 mM), the normalized spikelet number was 62.5 ± 7.6% of baseline (ACSF: 100.0 ± 4.9%, *n* = 8 cells, *P* = 0.002 vs. D-APV group; Figure 4B) and the AUC of the CS was 64.2 ± 7.4% of baseline (ACSF: 100.1 ± 6.6%, *n* = 8 cells, *P* = 0.002 vs. D-APV group; Figure 4C). These results indicate that the ethanol-induced suppression of the spontaneous CS waveform is not mediated by the NMDAR.

### The Ethanol-Induced Inhibition of CS Activity Is Abolished by CB1 Receptor Antagonism

**Figure 5:**
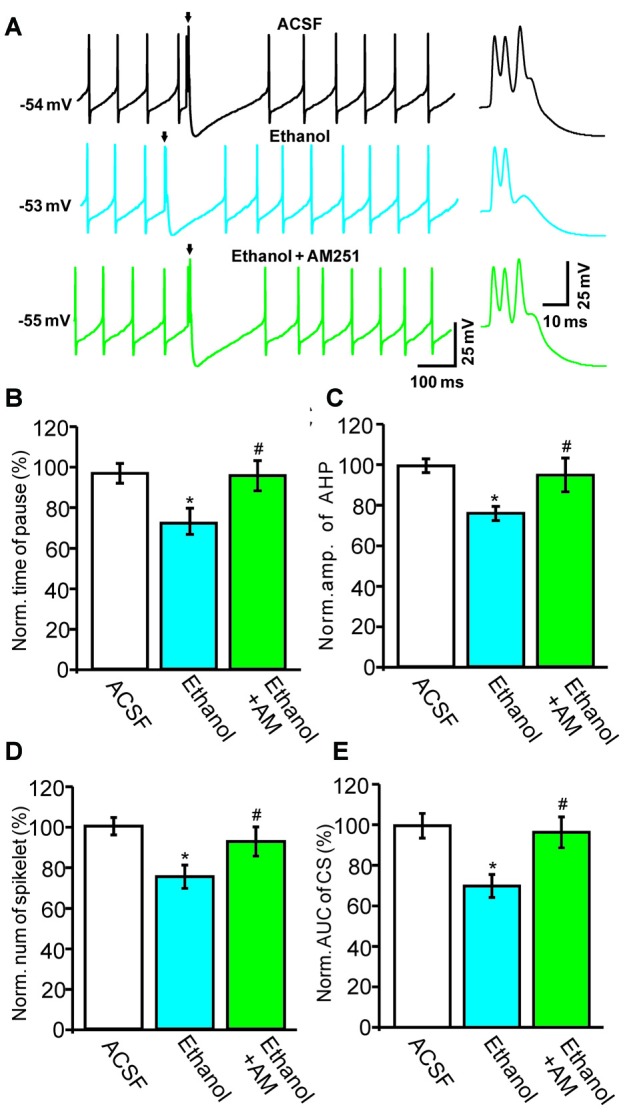
**Blockade of the cannabinoid receptor 1 (CB1) abolishes ethanol-induced inhibition of spontaneous CS activity. (A)** Representative traces showing the spontaneous activities of a PC in treatments of ACSF, ethanol (300 mM) and ethanol + AM251 (5 μM). Arrows denote CSs. **(B)** Pooled data showing the pause in SSs in treatments of ACSF, ethanol and ethanol + AM251. **(C)** Summary of data showing the normalized amplitude of the AHP in treatments of ACSF, ethanol and ethanol + AM251. **(D,E)** Bar graphs showing the effect of ethanol on the normalized spikelet number **(D)** and AUC **(E)** of CSs in treatments of ACSF, ethanol and ethanol + AM251. Arrows indicate spontaneous CSs. *n* = 6 cells per group. *P < 0.05, vs. ACSF; ^#^*P* < 0.05, vs. ethanol.

Next, we used a CB1 receptor antagonist, AM251, to evaluate the role of the CB1 receptor in the ethanol-induced inhibition of spontaneous CS activity in cerebellar PCs. Under current-clamp conditions (*I* = 0), ethanol produced a significant reduction in the pause in SS firing and in the amplitude of the AHP (Figure 5). Co-application of AM251 prevented this ethanol-induced inhibition of CS activity. In the presence of a mixture of ethanol and AM251, the normalized pause in SS firing was 98.7 ± 7.4% of baseline (ACSF: 100.0 ± 5.0%, *n* = 6 cells, *P* = 0.017 vs. ethanol alone: 75.4 ± 6.7% of baseline; Figures 5A,B) and the normalized amplitude of the AHP was 96.4 ± 8.1% of baseline (ACSF: 100.0 ± 3.7%, *n* = 6 cells, *P* = 0.021 vs. ethanol alone: 76.3 ± 3.8% of baseline; Figures 5A,C). In addition, application of AM251 abrogated the change in CS spikelets and the AUC of CSs to 93.2 ± 7.4% of baseline (100.0 ± 4.3%, *n* = 6 cells, *P* = 0.02 vs. ethanol alone: 76.2 ± 6.3% of baseline; Figures 5A,D) and 93.4 ± 7.6% of baseline (ACSF: 99.9 ± 6.0%, *n* = 6 cells, *P* = 0.016 vs. ethanol alone: 69.4 ± 5.6% of baseline; Figures 5A,E), respectively.

**Figure 6:**
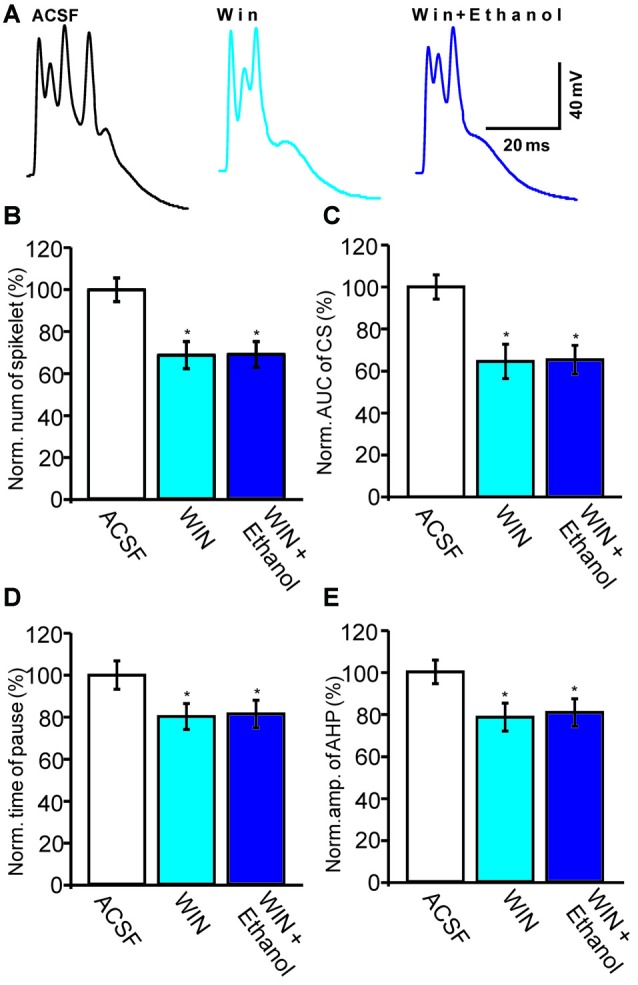
**Ethanol fails to inhibit the CS-evoked change in the membrane potential of PCs in the presence of a CB1 receptor agonist, WIN55212-2. (A)** Representative traces showing the spontaneous CS-evoked membrane potential in treatments of ACSF, WIN55212-2 (5 μM) and ethanol + WIN55212-2. **(B,C)** Bar graphs showing the normalized spikelet number **(B)** and AUC **(C)** of the CS-evoked potential in treatments of ACSF, WIN55212-2 and ethanol + WIN55212-2. **(D)** Pooled data showing the normalized pause in SSs in treatments of ACSF, WIN55212-2 and ethanol + WIN55212-2. **(E)** Bar graph showing the normalized amplitude of the AHP in treatments of ACSF WIN.55212-2 and ethanol + WIN55212-2. *n* = 6 cells per group. *P < 0.05, vs. ACSF.

Subsequently, we examined the effect of a CB1 agonist, WIN55212-2, on the ethanol-induced inhibition of CS activity. In the presence of WIN55212-2 (5 μM), the number of spikelets, the AUC of CSs, the pause in SS firing and the amplitude of the AHP were 68.7 ± 6.5% of baseline (ACSF: 100.0 ± 5.6%, *n* = 6 cells, *P* = 0.0003; Figures 6A,B), 64.5 ± 8.2% of baseline (ACSF: 100.0 ± 5.8%, *n* = 6 cells, *P* < 0.001; Figures 6A,C), 80.2 ± 6.2% of baseline (ACSF: 100.0 ± 6.8%, *n* = 6 cells, *P* = 0.0007; Figures 6A,D) and 78.4 ± 6.7% of baseline (ACSF: 100.0 ± 5.6%, *n* = 6 cells, *P* < 0.001; Figures 6A,E), respectively. These results indicate that activation of CB1 receptors inhibits spontaneous CS activity.

Interestingly, the pharmacological activation of the CB1 receptor prevented the ethanol (300 mM)-induced inhibition of CS activity. In the presence of a combination of WIN55212-2 and ethanol, the normalized number of spikelets was 69.1 ± 6.2% of baseline (*n* = 6 cells, *P* = 0.76 vs. WIN55212-2 alone; Figures 6A,B) and the normalized AUC was 65.3 ± 6.9% of baseline (*n* = 6 cells, *P* = 0.81 vs. WIN55212-2 alone; Figures 6A,C). In addition, the pause in SS firing was 81.4 ± 6.6% of baseline (*n* = 6 cells, *P* = 0.82 vs. WIN55212-2 alone; Figures 6A,E) and the normalized amplitude of the AHP was 80.6 ± 6.6% of baseline (*n* = 6 cells, *P* = 0.76 vs. WIN55212-2 alone; Figures 6A,D). These results indicate that pharmacological activation of CB1 receptors significantly depresses the spontaneous activity of CSs and prevents the ethanol-induced inhibition of the activity of spontaneous CSs.

## Discussion

In the present study, we demonstrated that ethanol dose-dependently depresses spontaneous CS activity in PCs—the alcohol decreased the AUC, the number of spikelets and the amplitude of the AHP. This ethanol-induced suppression of spontaneous CSs was abolished by a CB1 antagonist, but not by an NMDAR blocker. These findings suggest that ethanol inhibits spontaneous CS activity in PCs via activation of CB1 receptors *in vivo* in mice.

### Ethanol Modulates Spontaneous CS Activity in Cerebellar PCs

In the cerebellar cortex, AMPA receptors mediate the CS firing evoked by CF activity (Konnerth et al., 1990), while NMDARs contribute to CS firing by enhancing CS-evoked calcium transients (Piochon et al., 2007; Renzi et al., 2007; Liu et al., 2016). In addition, the NMDAR is a major target of ethanol in the brain, and has been implicated in ethanol-associated tolerance, dependance, withdrawal and relapse (Pignataro et al., 2009; Chandrasekar, 2013). It has been demonstrated that ethanol inhibits NMDAR-mediated excitatory postsynaptic responses in the hippocampus (Kolb et al., 2005) and dorsal striatum (Yin et al., 2007) *in vitro*. Recently, it was reported that ethanol decreases the amplitude of the afterdepolarization via activation of NMDARs in cerebellar slices (He et al., 2013). Indeed, our present results show that blocking the activity of NMDARs significantly decreases the number of spikelets and the AUC of CSs, suggesting that NMDARs contribute to spontaneous CSs. However, NMDAR blockade failed to prevent the ethanol-induced inhibition of CS activity, suggesting that the NMDAR is not involved in the ethanol-induced inhibition of spontaneous CS activity.

CF discharge is assumed to control the frequency and pattern of PC SS output by modulating tonic activity with variable-duration pauses. High frequency CS firing decreases the frequency of SS discharge (Ebner et al., 1983; Simpson et al., 1996; Cerminara and Rawson, 2004). In this study, we found that cerebellar surface application of ethanol induced a significant decrease in the pause and AHP amplitude, without changing the frequency of spontaneous SSs. This suggests that while ethanol modulates CS activity, manifested as decreases in the pause and amplitude of the AHP, it is incapable of significantly changing the SS firing rate in PCs. Our results are in general agreement with both *in vivo* and *in vitro* studies (Ming et al., 2006; Mameli et al., 2008), which suggest that ethanol does not substantially affect the spontaneous SS firing rate in PCs.

### Activation of CB1 Receptors Inhibits Synaptic Transmission at CF–PC Synapses

Endocannabinoids (eCBs) are released from PCs and MLIs upon activation of PF and/or CF inputs *in vitro* (Beierlein and Regehr, 2006; Soler-Llavina and Sabatini, 2006) and *in vivo* (Safo et al., 2006). It has been reported that activation of the CB1 receptor reduces neurotransmitter release from CFs onto PCs, suggesting that the eCB system is involved in the modulation of CF–PC synaptic transmission (Maejima et al., 2001). Kawamura et al. (2006) demonstrated that the CB1 receptor is the major cannabinoid receptor at presynaptic sites of CF–PC synapses, and that pharmacological activation of the CB1 receptor inhibits excitatory glutamate release from CF–PC synapses, resulting in a depression of EPSCs evoked by CS activity under *in vitro* conditions (Kawamura et al., 2006). We recently found that repeated facial stimulation evokes eCB release from MLIs via activation of NMDARs, which is involved in MLI–PC GABAergic synaptic LTD (Bing et al., 2015). In the present study, application of a CB1 receptor agonist induced a significant inhibition of CF–PC synaptic transmission, evidenced by decreases in the number of spikelets and the AUC of CSs. This finding is also consistent with previous studies (Maejima et al., 2001; Kawamura et al., 2006), which indicate that activation of the CB1 receptor inhibits excitatory transmitter release at CF–PC synapses.

### Ethanol Modulates the Spontaneous CS Activity of PCs via Activation of CB1 Receptors

In the current study, ethanol-induced inhibition of CS activity was prevented by extracellular application of a CB1 receptor antagonist, AM-251. This suggests that ethanol suppresses spontaneous CS activity *in vivo* by activating the CB1 receptor. This observation is in line with previous studies (Basavarajappa et al., 2008; Perra et al., 2008) showing that ethanol inhibits CS activity via activation of presynaptic CB1 receptors at CF–PC synapses.

Ethanol enhances eCB levels through calcium pathways and depresses miniature postsynaptic current frequencies via activation of CB1 receptors in cultured hippocampal neurons, suggesting that eCBs function as retrograde messengers in the ethanol-induced depression of synaptic activities (Basavarajappa et al., 2008). Furthermore, ethanol inhibits the spontaneous activity of pyramidal neurons in the basolateral nucleus of the amygdala by activating the eCB system *in vivo* in rats (Perra et al., 2008). Moreover, pharmacological activation of CB1 blocks the ethanol-induced increase in the frequency of spontaneous inhibitory postsynaptic currents in cerebellar PCs (Kelm et al., 2008), and is sufficient to prevent the ethanol-induced presynaptic facilitation of GABAergic signaling onto pyramidal neurons in the central amygdala (Roberto et al., 2010). Our present findings also show that CB1 receptor agonism not only significantly inhibits CS activity, it also prevents the ethanol-induced depression of CSs, suggesting that ethanol depresses CSs by activating the CB1 receptor. In addition, it has been demonstrated that ethanol prevents the eCB-mediated long-lasting disinhibition of striatal output induced by electrical stimulation, but not by CB1 receptor agonism, suggesting that ethanol affects CB1 receptor-mediated signaling in a synapse-specific manner (Clarke and Adermark, 2010).

CS activity is thought to help trigger synaptic plasticity in the cerebellar cortex, thereby contributing to motor learning. The induction of PF–PC synaptic LTD requires co-activation of the PF and CF inputs, suggesting that CS activity may play a critical role in motor learning (Hansel et al., 2001; Ito, 2001). Our results show that ethanol significantly depresses CS activity in PCs by modulating the CB1 receptor, suggesting that it might also affect the induction of cerebellar PF–PC LTD. Indeed, it was demonstrated that ethanol impairs the PF LTD induced by co-activation of PFs and CFs, without impairing LTD induced by PF stimulation in cerebellar slices (He et al., 2013). Moreover, ethanol inhibits NMDAR-dependent LTP at glutamatergic synapses onto medium spiny dorsal striatal neurons via the CB1 receptor in the hippocampus (Yin et al., 2007). The effect of ethanol on outward currents might be influenced by urethane anesthesia. However, urethane depresses neuronal excitability through activation of barium-sensitive potassium leak conductance, without affecting glutamate-mediated excitatory synaptic transmission or GABA_A_/B-mediated inhibitory synaptic transmission (Sceniak and Maciver, 2006).

In conclusion, our findings show that ethanol dose-dependently inhibits spontaneous CS activity by activating CB1 receptors *in vivo*. This suggests that excessive ethanol intake might affect PF–PC synaptic plasticity and motor learning by inhibiting CS activity via CB1 receptors at CF–PC synapses.

## Author Contributions

C-PC, S-BC and D-LQ conceived and designed the experiments; G-JZ, M-CW and J-DS performed the experiments; C-PC and D-LQ analyzed the data; Y-HX contributed reagents/materials/analysis tools; C-PC and D-LQ wrote the manuscript. All authors listed, have made substantial, direct and intellectual contribution to the work and approved it for publication.

## Conflict of Interest Statement

The authors declare that the research was conducted in the absence of any commercial or financial relationships that could be construed as a potential conflict of interest.
